# Nursing staff’s attitudes towards the prevention of adverse events among hospitalized people with dementia: Protocol of qualitative systematic review and evidence synthesis

**DOI:** 10.1371/journal.pone.0301651

**Published:** 2024-09-09

**Authors:** Lucía Catalán, Anne Margriet Pot, Amy Pepper, Karen Harrison Dening, Déborah Oliveira

**Affiliations:** 1 Faculty of Healthcare Sciences, Universidad San Sebastián, Santiago, Chile; 2 Faculty of Nursing, Universidad Andrés Bello, Santiago, Chile; 3 Millenium Institute for Care Research (MICARE), Santiago, Chile; 4 Erasmus School on Health Policy & Management, Erasmus University Rotterdam, Rotterdam, The Netherlands; 5 Optentia, North-West University, Vanderbijlpark, South Africa; 6 HC-One, Darlington, United Kingdom; 7 Dementia UK, London, United Kingdom; 8 School of Nursing and Midwifery, De Montfort University, Leicester, United Kingdom; Utah State University, UNITED STATES OF AMERICA

## Abstract

**Introduction:**

People with dementia are more likely than people without dementia to be hospitalized and to experience in-hospital preventable adverse events, such as falls, skin injury, and infection, compared to other hospitalized groups. Negative attitudes towards people with dementia are common among acute healthcare workers and have been linked to a cascade of negative adverse events in this population. However, no qualitative systematic review has ever been conducted to synthesize the existing evidence in this area, which hampers the development of preventative measures.

**Aim:**

This is a protocol for a qualitative systematic review aimed at exploring and synthesizing existing qualitative evidence regarding the attitudes of nursing staff towards the prevention of adverse events among hospitalized people with dementia.

**Methods:**

Literature searches will be performed in PubMed, CINAHL, PsycINFO, Web of Science, Biblioteca Virtual de Salud, Scopus, The Cochrane Library, and Google Scholar. The references of eligible studies will be checked for eligibility. All primary qualitative or mixed-methods studies with a qualitative component published in peer-reviewed academic journals in English, Portuguese, or Spanish will be eligible. There will be no limitations to the date of publication. The selection process will be conducted independently by two researchers using the software Rayyan and then compared and discussed. Any disagreements regarding eligibility will be discussed among the entire research team and resolved via consensus. Methodological quality will be assessed using Cochrane’s guidance. A meta-aggregative approach will be employed to extract and synthesize the evidence using the software package QARI from the JBI. The confidence in the findings will be graded using ConQual.

**Implications:**

This review will help identify and better understand specific attitudinal and psychosocial aspects that influence nursing care delivery for people with dementia in hospital settings. Such data can be used to generate novel explanatory models of nursing behaviors in dementia care, as well as capacity building and training to enhance hospital care for people with dementia globally.

## Introduction

There are over 55 million people with dementia worldwide and this number is expected to triple by 2050 [1]. People with dementia are more likely than people without dementia to be hospitalized, and the unfamiliar surroundings and the one-size-fits-all approaches and routines within hospital environments can be particularly challenging for such individuals [2]. Hospital-based adverse events (AE) are common among people with dementia, such as falls, pressure ulcers, delirium, unmanaged pain, medication errors, undernutrition, infections, hospital-acquired incontinence, and decline in individual capacities [3, 4]. These are linked to longer hospital stays, higher healthcare costs, higher risk of transitioning into a care facility, and increased mortality [5].

Such AE and other related negative outcomes can be partly explained by the high rates of multimorbidity and polypharmacy among people with dementia, but poor practice related to healthcare workers’ attitudes towards people with dementia also a role [6, 7]. An attitude constitutes the way we see, think, and feel towards a person, group, condition, or an object, reflecting our views, beliefs, knowledge, emotions, and past experiences associated with those, on a dimension ranging from negative to positive [8]. In healthcare, examples of positive attitudes include being nonjudgmental and attentive to a person’s needs, being empathetic, and expressing positive views about that person. On the other hand, examples of negative attitudes include being unable to see the person behind a diagnosis or avoiding contact with a person due to holding negative views about that person’s condition.

Many studies have highlighted the key role that healthcare workers’ positive attitudes play on the effective prevention of AE in various other settings and population groups [e.g., 9–11]. In dementia, attitudes of healthcare workers are generally assessed in relation to people with dementia and their care more generally, rather than to the safety of their care [12–14]. Overall, negative attributes and stereotypes towards older people and people with dementia are common among acute healthcare workers [15] and include considering people with dementia as incapable of being involved in their care decisions [16], as “challenging” or “difficult patients” [17], and as invariably dependent of care [4]. Some researchers have linked these to a cascade of negative events, such as hospital-acquired incontinence, physical and emotional harm, and death [3]. A recent study demonstrated a significantly inverse relationship between nurses’ attitudes towards patient safety and missed nursing care, which may contribute to an increased occurrence of AE [18]. In other studies in which the prevalence and types of AE affecting people with dementia are explored, “dementia” is considered as a risk factor, disregarding the role of a multitude of external factors in producing such events [19]. In other studies, AE and negative outcomes affecting people with dementia are “justified” as being caused by the person’s poor behavior, their “poor cooperation” or their “inability to understand” instructions [20, 21], which inadvertently puts the responsibility of malpractice on the person and their dementia and overshadows their needs. It is also possible that healthcare workers hold contradicting attitudes towards patient safety, which can influence care outcomes. For example, some data suggest that healthcare workers can perceive patient safety as a priority at the same time as they express fear of reporting errors due to possible retaliations or inaction from managers [22]. In dementia, it might be that nursing staff’s negative attitudes towards people with dementia could contribute to underreporting as workers may feel the event might have been inevitable, or that the person with dementia likely contributed to it.

Systematic reviews and synthesis of qualitative evidence are important to inform evidence-based healthcare. Previous reviews have synthesized qualitative evidence regarding healthcare workers’ experiences in caring for people with dementia in hospital settings [17], nurses’ views about facilitators of person-centered care for inpatients living with dementia [23], existing knowledge of dementia-friendly hospital design [24], the effectiveness of specific interventions in improving hospital safety for people with dementia [25], and attitudes towards specific negative practices such as physical restraints [26]. However, a review of nursing staff’s attitudes towards the prevention of adverse events in people with dementia who are hospitalized has never been conducted. Such review can help identify and better understand specific attitudinal and psychosocial aspects that influence nursing care delivery for people with dementia in hospital settings. Such knowledge can be used to generate novel explanatory models of nursing behaviors in dementia care, as well as capacity building and training initiatives to enhance hospital care for people with dementia globally.

## Aim

This systematic review is aimed at exploring and synthesizing the existing qualitative evidence regarding the attitudes of nursing staff towards the prevention of AE among people with dementia who are hospitalized.

## Methods

The review will be conducted in line with the current methodological guidance for qualitative systematic reviews [27] and reported as per the PRISMA statement [28] and the ENTREQ framework for qualitative synthesis [29]. The review protocol was registered in PROSPERO (CRD42023488760) and is hereafter presented in line with PRISMA-P (S1 Appendix) [30].

### Review question

What are the nursing staff’s attitudes towards the prevention of AE in people with dementia in hospital settings?

### Search strategy

The search strategy was built using the PerSPE(C)TiF framework, which is appropriate for qualitative systematic reviews (**S2 Appendix**). Relevant search terms in English, Portuguese, and Spanish were identified in the MeSH and DeCS databases, and in key literature reviews related to the area of interest. The search was tailored to identify research that is specific to AE that occur more frequently among people with dementia in hospital settings (e.g., falls, pressure ulcers, hospital-acquired incontinence, untreated pain, undernutrition, and decline in physical and cognitive capacity).

### Eligibility criteria

The review will include studies focused on exploring the views, experiences, and/or attitudes of nursing staff (i.e., nurses, nursing technicians, auxiliary nurses) regarding care safety and the prevention of any AE in people with dementia being cared for in any hospital/ acute setting/unit. All primary qualitative or mixed-methods studies with a qualitative component published in English, Portuguese, or Spanish will be eligible for inclusion as the authors are fluent in these and they are major idioms in the world. Primary studies published in peer-reviewed academic journals, as well as theses, dissertations, and conference abstracts will be eligible. All publications available from the start date of each research database will be eligible as this will give us the opportunity to explore whether nurses’ attitudes appear to have changed over time. Studies conducted with healthcare students, and studies that do not specifically mention the population of people with dementia (e.g., only referred to cognitive impairment or memory loss) will be excluded. Editorials, personal views, and case reports will also be excluded.

### Literature search and study selection

First, literature searches will be performed in PubMed (NCBI), CINAHL (EBSCOhost), PsycINFO (EBSCOhost), Web of Science (Clarivate), Biblioteca Virtual de Salud (BVS), Scopus (Elsevier), The Cochrane Library, and Google Scholar. All retrieved documents will be exported to the Rayyan platform for deletion of duplicates and study selection, first based on the content of titles and abstracts and then upon reading their full content. The references of included studies will also be checked for inclusion of any eligible publication. The entire selection process will be conducted by two researchers independently (LC, AP) and then compared and discussed by the entire research team (DO, LC, AP, KHD, AMP). Any disagreements regarding eligibility will be resolved via consensus. Eligible studies will then be assessed in terms of methodological quality.

### Methodological quality

While we acknowledge that researchers should not simplistically adopt any existing quantitatively informed instruments to appraise qualitative research without considering each qualitative research nuances and idiosyncrasies, we consider it crucial that some level of critical appraisal is undertaken to assess the trustworthiness of the existing evidence to allow for future informed decisions regarding knowledge generation. Accordingly, Cochrane’s guidance will be used to assess the methodological limitations of the eligible studies [31] without excluding any of them from this evaluation. Instead, the results of this evaluation will be reported transparently and discussed thoroughly in the review report, with a detailed consideration of their internal and external validity. The appraisal process will be conducted independently by two researchers (DO, KHD), and then compared and discussed among the entire research team (DO, LC, AP, KHD, AMP). Any disagreements regarding quality scores will be resolved by consensus among the entire research team.

### Data extraction and qualitative synthesis

A meta-aggregative approach will be employed to extract and synthesize the evidence as presented in the included studies, avoiding any re-interpretation. Meta-aggregation is rooted in pragmatism and has a process-driven approach with the ultimate goal of producing findings that can inform healthcare policies and practices. The recommendations arising from a meta-aggregative review are deemed practical, specific, detailed, and measurable because of the transparent link to the data as described in the included studies [32].

The key steps of such method, as proposed by Lockwood et al. [33], are as follows. First, key information about each included study will be extracted using an extraction form and will include the study reference, phenomenon of interest, sample and setting characteristics, and study design and methods. Qualitative findings will consist of any verbatim extract of the author’s analytical interpretation of the results or data and will be extracted together with an illustration from the same study that informs the finding (e.g., a direct quotation of the participant’s voice, fieldwork observations, or any other supporting data). For mixed-methods studies, only the findings related to their qualitative components will be extracted.

For each finding and its accompanying illustration, a level of plausibility will be established as follows: I. Unequivocal: it is beyond reasonable doubt, and therefore not open to challenge; II. Equivocal: It lacks clear association with it and is therefore open to challenge; and III. Unsupported: This is not supported by the data. Findings classified as III will not be included in the review findings but will be reported separately for transparency. The software package QARI from the Joanna Briggs Institute will be used to synthesize the extracted data and will consist of two parts: I. Development of categories for findings with at least two findings per category; and II. Development of one or more synthesized findings from at least two categories.

### Assessment of confidence in the findings

The final synthesized findings will be graded according to the ConQual approach for establishing confidence in the output of qualitative research synthesis and presented as a Summary of Findings [34]. Each synthesized finding from the review will then be presented and discussed along with the type of research informing it, the score for dependability and credibility, and the overall ConQual score.

## Discussion

Healthcare staff attitudes towards patient care have a significant impact on the quality of care provided, influencing patients’ outcomes, wellbeing, and recovery. Studies have shown that nursing staff often have negative attitudes towards hospitalized people with dementia and their care. This is the first systematic review to thoroughly explore and synthesize qualitative evidence about the attitudes of nursing staff toward the prevention of adverse events with people with dementia in hospitals. An international and multidisciplinary team of researchers will be involved and will follow robust strategies for evidence review and synthesis, which will then be appraised and reported transparently. The goal is to achieve the highest possible confidence in the evidence, whilst also considering different approaches and nuances related to each researchers’ contexts and experiences through a reflexive and collaborative approach. The results have the potential to inform quality improvement strategies in nursing care for this population, which will likely contribute to reducing the current negative outcomes shown in several studies with such individuals.

Nursing staffs’ attitudes are probably highly contextual and influenced by numerous external and broader factors, such as individual cultures and cultures of care, services and systems’ structures, and professional training and roles. We anticipate that most of the existing evidence will come from high-income nations, and therefore will likely not be representative from low-income and culturally and ethnically diverse contexts. We hope our broader idiom and database search will help the review be less limited to Western and English-speaking countries like most reviews are. We will also be inclusive of dissertations and theses that have not been published, helping gather evidence from studies from low-income contexts which could not be published due to limited access (i.e., paywall / publication fees). We anticipate slight changes will need to be made to our search strategy as we advance in the review process and adapt to the various database requirements. Such changes will be reported transparently together with the review results.

## Supporting information

S1 Appendix PRISMA-ScR. (DOCX)

S2 Appendix PerSPE(C)TiF search strategy. (DOCX)
