# Correction: Nursing staff’s attitudes towards the prevention of adverse events among hospitalized people with dementia: Protocol of qualitative systematic review and evidence synthesis

**DOI:** 10.1371/journal.pone.0321865

**Published:** 2025-04-02

**Authors:** Lucía Catalán, Anne Margriet Pot, Amy Pepper, Karen Harrison Dening, Déborah Oliveira

The following information is missing from the Funding statement: This work is supported by the Virrectoría de Investigación y Doctorados de la Universidad San Sebastián - Fondo USS-FIN-24-APCS-24.
