# Supplementary material for: Nursing staff’s attitudes towards the prevention of adverse events among hospitalized people with dementia: Protocol of qualitative systematic review and evidence synthesis
Source: PLoS One. 2024 Sep 9;19(9):e0301651. doi: 10.1371/journal.pone.0301651 (PMC11383228; doi:10.1371/journal.pone.0301651)
Supplement: S2 Appendix — (DOCX) [file pone.0301651.s002.docx]

**APPENDIX II**

| **PerSPE(C)TiF search strategy (Nursing staff’s attitudes towards the prevention of adverse events among people living with dementia in hospitals: Protocol of systematic review and synthesis of qualitative evidence)** | | | | | | | | | | |
| --- | --- | --- | --- | --- | --- | --- | --- | --- | --- | --- |
|  | **Per** | **S** | **P** | | **E** | | **(C)** | **Ti** | | **F** |
| **PerSPE(C)TiF** | **Perspective** | **Setting** | | **Phenomenon of interest / Problem** | **Environment** | | **Comparison (optional)** | **Time/Timing** | | **Findings** |
|  | Nursing staff | Hospitals or other acute care settings | | Care safety / Prevention of AE in PLWD | Any hospital unit | | With or without a comparison group (any) | Period of hospitalization | | Attitudes |
| **MeSH** | | | | | | | | | | |
| **OR** | “inpatients” [MeSH Terms] “hospitalization” [MeSH Terms]  “hospitals” [MeSH Terms]  “tertiary care centers” [MeSH Terms]  “hospital medicine” [MeSH Terms]  “hospital units” [MeSH Terms]  “hospitals, general” [MeSH Terms]  “hospital*” [All Fields]  “hospital personnel” [MeSH Terms]  “nursing staff, hospital” [MeSH Terms]  “nursing service, hospital” [MeSH Terms]  “medical staff, hospital” [MeSH Terms]  “hospitalisation*” [All Fields]  “hospitalization*” [All Fields]  “hospitalize*” [All Fields]  “hospitalise*” [All Fields]  “hospital care” [All Fields]  “hospital” [All Fields]  “impatient*” [All Fields]  “impatient care” [All Fields] | | | “patient harm” [MeSH]  “medical errors” [MeSH]  “medication errors” [MeSH]  “near miss, healthcare” [MeSH]  “diagnostic errors"[MeSH]  “pressure ulcer” [MeSH]  “accidental falls” [MeSH]  “patient safety” [MeSH]  “risk management” [MeSH]  “safety management” [MeSH]  “delirium” [MeSH]  “patient safety” [All Fields]  “care safety” [All Fields]  “hospital care safety” [All Fields]  “risk management” [All Fields]  “safety management” [All Fields]  “patient harm” [All Fields]  “medication error*” [All Fields]  “drug use error*” [All Fields]  “healthcare near miss” [All Fields]  “patient incident*” [All Fields]  “adverse event*” [All Fields]  “risk averse” [All Fields]  “medical error*” [All Fields]  “diagnostic error*” [All Fields]  “pressure ulcer*” [All Fields]  “fall*” [All Fields]  “adverse outcome*” [All Fields]  “deliri*” [All Fields]  “harm*” [All Fields] | | “dementia” [MeSH Terms]  “dementia vascular” [MeSH Terms]  “frontotemporal dementia” [MeSH Terms]  “Alzheimer disease” [MeSH Terms]  “mixed dementias” [MeSH Terms]  “Lewy body dementia” [MeSH Terms]  “dement*” [All Fields]  “Alzheimer*” [All Fields]  “people with dementia” [All Fields]  “people living with dementia” [All Fields]  “person with dementia” [All Fields]  “person living with dementia” [All Fields]  “patients with dementia” [All Fields]  “patients living with dementia” [All Fields]  “patient with dementia” [All Fields]  “patient living with dementia” [All Fields] | | | “attitude” [MeSH Terms]  “attitude of health personnel” [MeSH Terms]  “knowledge” [MeSH Terms]  “attitude*” [All Fields]  “knowledge*” [All Fields]  “views and attitudes” [All Fields]  “belief*” [All Fields] | |
| **DeCS Spanish** | | | | | | | | | | |
| **OR** | ”pacientes internos” [DECs]  ” hospitalización” [DECs]  ”hospitales” [DECs]  “centros de atención terciaria” [DECs]  “medicina hospitalar” [DECs]  “unidades hospitalarias” [DECs]  “hospitales generales” [DECs]  “personal de hospital” [DECs]  “personal de enfermería” [DECs]  “personal de enfermería en hospital” [DECs]  “cuerpo médico” [DECs]  “paciente* hospitalizado*” [All Fields]  “cliente*” [All Fields]  “hospital*” [All Fields]  “clinica*” [All Fields] | | | “daño del paciente” [DECs]  “errores médicos” [DECs]  “errores de medicación” [DECs]  “near miss salud” [DECs]  “errores diagnósticos” [DECs]  “accidentes por caídas” [DECs]  "úlcera por presión" [DECs]  "seguridad del paciente" [DECs]  “gestión de riesgos” [DECs]  “administración de la seguridad” [DECs]  “delirio” [DECs]  “deliri*” [All Fields]  "seguridad del paciente" [All Fields]  "gestión de riesgos" [All Fields]  “gestión de la seguridad” [All Fields]  "daño al paciente" [All Fields]  incidente* [All Fields]  "evento* adverso*" [All Fields]  “evitación de riesgo*” [All Fields]  "near miss" [All Fields]  "error* médico*" [All Fields]  “error* diagnóstico*” [All Fields]  "error* de medicación" [All Fields]  “úlcera* por presión” [All Fields]  caída* [All Fields]  "resultado* adverso*” [All Fields] | | "enfermedad de Alzheimer" [DECs]  "demencia" [DECs]  “demencia frontotemporal” [DECs]  “demencias mixtas” [DECs]  “enfermedad por cuerpos de Lewy” [DECs]  "demencia vascular" [DECs]  “demen*” [All Fields]  “alzheimer*” [All Fields]  “paciente* viviendo con demencia” [All Fields]  “persona* viviendo con demencia” [All Fields]  “paciente* con demencia” [All Fields]  “persona* con demencia” [All Fields] | | | “actitud del personal de salud” [DECs]  “actitud” [DECs]  “conocimiento” [DECs]  “actitud*” [All Fields]  “conocimiento” [All Fields]  “visión y actitud*” [All Fields]  “creencia*” [All Fields] | |
|  | **AND** | | | | | | | | | |
| **DeCS Portuguese** | | | | | | | | | | |
| **OR** | “hospitalização” [DECs]  “pacientes internados” [DECs]  “hospitais” [DECs]  “centros de atenção terciária” [DECs]  “unidades hospitalares” [DECs]  “medicina hospitalar” [DECs]  “hospitais gerais” [DECs]  “recursos humanos em hospital” [DECs]  “recursos humanos de enfermagem” [DECs]  “corpo clínico” [DECs]  “paciente hospitalizado” [All Fields]  “hospita*” [All Fields]  “clinic*” [All Fields] | | | “delírio” [DECs]  “danos ao paciente” [DECs]  “erros médicos” [DECs]  “erros de medicação” [DECs]  “near miss” [DECs]  “erros de diagnóstico” [DECs]  “acidentes por quedas” [DECs]  " lesão por pressão " [DECs]  " segurança do paciente " [DECs]  “gestão de riscos” [DECs]  “gestão da segurança” [DECs]  “delíri*” [All Fields]  “segurança do paciente” [All Fields]  “gerenciamento de riscos” [All Fields]  “gerenciamento de segurança” [All Fields]  “danos ao paciente” [All Fields]  “erros de medicação” [All Fields]  “quase acidente” [All Fields]  “incidente*” [All Fields]  “prevenção de risco” [All Fields]  “evento adverso” [All Fields]  “erro médico*” [All Fields]  “erro de diagnóstico” [All Fields]  “úlcera por pressão” [All Fields]  “queda*” [All Fields] | | “demência” [DECs]  "doença de Alzheimer" [DECs]  "demência" [DECs]  "demência vascular" [DECs]  “demência frontotemporal” [DECs]  “demências mistas” [DECs]  “doença por corpos de Lewy” [DECs]  “demência*” [All Fields]  “Alzheimer*” [All Fields] | | | “atitude do pessoal de saúde” [DECs]  “atitude” [DECs]  “conhecimento” [DECs]  “atitud*” [All Fields]  “visão *” [All Fields]  “crença*” [All Fields]  “conhecimento*” [All Fields] | |
|  | **AND** | | | | | | | | | |
